# Bone metastasis manifested 52 years after resection of an apparently benign paraganglioma: A case report

**DOI:** 10.1177/2050313X241229853

**Published:** 2024-02-07

**Authors:** Run Yu, Martin S Auerbach, Nathan S Honda

**Affiliations:** 1 Division of Endocrinology, UCLA David Geffen School of Medicine, Los Angeles, CA, USA; 2 Department of Nuclear Medicine and Department of Molecular and Medical Pharmacology, UCLA David Geffen School of Medicine, Los Angeles, CA, USA; 3 Department of Pathology, PIH Health, Whittier, CA, USA

**Keywords:** Paraganglioma, late metachronous metastasis, avulsion fracture, lesser trochanter

## Abstract

Paraganglioma is derived from the paraganglia tissue in the neck, along the sympathetic trunk, and in the pelvis. Paraganglioma has malignant potential and can metastasize to remote organs such as the liver, lungs, and bones. Most metachronous metastases occur within several years after the initial diagnosis of paraganglioma. Here, we report the case of a 71-year-old male patient who developed bony metastasis 52 years after the resection of a large paraganglioma at the aortic bifurcation. The biopsy-proven paraganglioma metastasis to the lesser trochanter of left femur presented as an avulsion fracture. His normetanephrine level was elevated. DOTATATE PET (positron emission tomography) did not find any other metastatic lesions. The bony metastasis was treated with radiation therapy. We believe that the patient had one of the longest gaps ever reported, 52 years, between the initial diagnosis and metastasis of paraganglioma. This case highlights the importance of long-term surveillance of patients with paraganglioma for metastasis.

## Introduction

Paraganglioma is a tumor derived from the paraganglia tissue in the neck, along the sympathetic trunk, and in the pelvis.^1,2^ Pheochromocytoma is a similar tumor, but it is derived from the adrenal medulla. Approximately 50% of paragangliomas secrete catecholamines which cause hypertension and paroxysmal palpitation, headache, and sweating.^1^ Paraganglioma has malignant potential and can metastasize to remote organs such as the liver, lungs, and bones.^3,4^ Paraganglioma metastasis can be synchronous (identified at the initial diagnosis of paraganglioma) in about one-third of patients or metachronous (identified after the initial diagnosis of paraganglioma) in about two-thirds of patients.^5^ Most metachronous metastases occur within several years after the initial diagnosis of paraganglioma.^5^ Late paraganglioma metastasis cases have been reported.^6–9^ Here, we report a case of paraganglioma, which we believe has one of the longest gaps ever reported, 52 years, between the initial diagnosis and metastasis. Our case highlights the importance of long-term surveillance of patients with paraganglioma for metastasis or recurrence.

## Case report

A 71-year-old male presented with progressive left hip pain. About 6 months prior to presentation, he had started experiencing left thigh pain and difficulty in weight bearing on the left low extremity. A few months later, he had experienced unintentional weight loss and lost 20 pounds prior to presentation.

**Figure 1.**
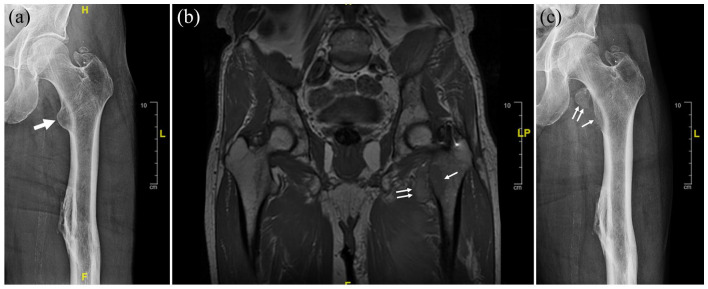
Avulsion fracture at the lesser trochanter of left femur. (a) X-ray of left hip 2 months before presentation. (b) MRI of left hip a few days after presentation showed avulsion fracture of the lesser trochanter of left femur with detachment of the iliopsoas muscle tendon from the left femur. (c) X-ray of the left hip 1 month after presentation. Thick arrow: lytic lesion on the lesser trochanter of left femur; thin arrow: remaining lytic lesion on the lesser trochanter of left femur after avulsion fracture. Double thin arrows: the detached lytic lesion from the lesser trochanter of left femur after avulsion fracture. Note the migration of the detached lesion 1 month after presentation.

His past medical history dated back to his teenage when he had headache and vomiting after exercise, and intermittent diaphoresis, hypertension, and tachycardia for a few years. At age 19, he was diagnosed with pheochromocytoma or paraganglioma based on elevated urine vanillylmandelic acid levels. The tumor was located by aortogram which revealed a 7-cm vascular mass at the aortic bifurcation. His symptoms completely disappeared after surgical removal of the paraganglioma, and he was told that the tumor was benign and did not require routine follow-up. The successful angiographic location of his paraganglioma was actually described in a case report published in 1974.^10^ At age 23, he had an accident which resulted in visceral trauma and left femoral shaft fracture and he underwent left nephrectomy and splenectomy. At age 58, he was found to harbor colon adenocarcinoma (T3N1M0) and received partial chemotherapy. He underwent surveillance for colon cancer recurrence or metastasis by CEA (carcinoembryonic antigen) testing every 3–6 months, CT (computed tomography) of abdomen and pelvis every 6–12 months, and colonoscopy every year. The surveillance did not find evidence of colon cancer prior to presentation. A left adrenal nodule was incidentally found and remained largely stable in size. Available outside medical records showed that the adrenal nodule had already been present 9 years before presentation, and that it was non-enhancing on CT and measured 1.6 cm × 1.3 cm. One and a half years later, the nodule size remained unchanged. At presentation, 9 years after the nodule was known, the nodule measured 2.5 cm × 1.8 cm and exhibited Hounsfield units under 10 on CT without contrast. The low pre-contrast Hounsfield units, lack of enhancement, and slow growth speed established the diagnosis of adrenal adenoma. At age 69, about 1.5 years prior to presentation, he gradually developed hypertension and began antihypertensive medications. X-ray of the left hip 2 months before presentation was reportedly unremarkable, but in retrospect, a subtle lytic lesion at the lesser trochanter of left femur was present (Figure 1).

**Figure 2.**
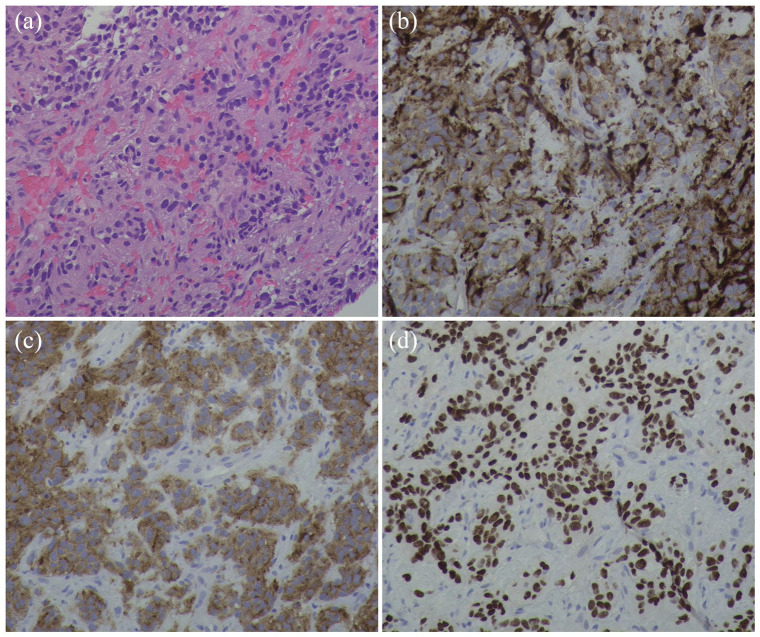
Histology of the core biopsy specimen from the left lesser trochanter. (a) Hematoxylin and eosin stain. (b) Chromogranin A immunostain. (c) Synaptophysin immunostain. (d) GATA3 immunostain. 200×.

**Figure 3.**
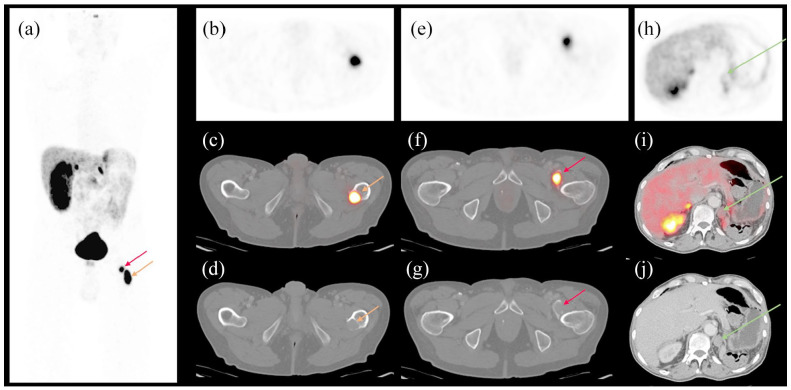
DOTATATE PET/CT 1 month after presentation. (a) Maximal intensity projection view. (b–d) Axial view of the remaining lytic lesion of the lesser trochanter of left femur (red arrows). (e–g) Axial view of the detached lytic lesion from the lesser trochanter of left femur (yellow arrows). (h–j) Axial view of the left adrenal nodule (green arrows). (b, e, and h) DOTATATE PET images. (c, f, and i) Overlay of PET and CT images. (d, g, and j) CT images.

At presentation, he denied paroxysmal headache or vomiting. His blood pressure had been well controlled by daily metoprolol 100 mg, losartan 100 mg, and amlodipine 5 mg. He had no family history of paraganglioma. He was retired. His sister and he were the only children of their parents, and each had no children. Physical examination showed antalgic gait due to left thigh pain without tenderness or swelling in the left hip or lower extremity. Plasma metanephrines were tested due to the recent-onset hypertension and showed metanephrine 0.26 nmol/L (range <0.49) and normetanephrine 7.64 nmol/L (<0.89). MRI (magnetic resonance imaging) of left hip showed avulsion fracture of the lesser trochanter of left femur with detachment of the iliopsoas muscle tendon from the left femur (Figure 1). Core biopsy of the left lesser trochanter was obtained under CT-guidance at an outside hospital. Sections of the specimens demonstrated a cellular malignant neoplasm with neuroendocrine histology and fine branching capillary channels (Figure 2). The tumor cells exhibited relatively uniform round to oval nuclei and finely dispersed chromatin arranged in a trabecular nested pattern. Immunostaining was positive for chromogranin A, synaptophysin, S100, and GATA3 (Figure 2). The overall morphologic and immunophenotypic features were consistent with metastatic paraganglioma. The patient was further evaluated by endocrinology, orthopedics, and radiation oncology. Doxazosin 2 mg was started, replacing losartan. Doxazosin dose was raised to 2 mg twice daily to better control the blood pressure. DOTATATE PET/CT showed pathologic avulsion fracture of the left lesser trochanter at the site of a lytic metastasis and intensely DOTATATE-avid tumor involving both fracture fragments (Figure 3). The left adrenal nodule did not exhibit DOTATATE signal. No sites of a primary tumor or other metastatic lesions were identified. He received external beam radiation to the left femur lesions at a dose of 3000 cGy over 10 fractions. He still had intermittent pain with weight bearing after the radiation therapy. The normetanephrine level decreased to 6.34 nmol/L 3 months after the completion of radiotherapy. His blood pressure remained well controlled by doxazosin, metoprolol, and amlodipine. The patient declined genetic testing.

## Discussion

In this report, we describe a patient with a very remote history (52 years before presentation) of resection of a large functional paraganglioma at the aortic bifurcation who developed left femur metastasis resulting in avulsion fracture. As the DOTATATE PET did not show any other potential primary sites of paraganglioma, the left femur metastatic paraganglioma most likely comes from the original paraganglioma at the aortic bifurcation. Our case highlights the importance of long-term surveillance of patients with paraganglioma for metastasis or recurrence.

Early reports on paragangliomas at the aortic bifurcation suggest that they usually exhibit an overall benign or at least indolent behavior,^11^ which was probably why the patient was told he had a benign tumor at age 19. Modern literature shows that as much as 50% of abdominal paragangliomas can be malignant and recommends life-long surveillance by biochemical tests and imaging for recurrence or metastasis.^1,3–5^ The diagnosis of malignant paraganglioma relies ultimately on whether the paraganglioma metastasizes to remote organs that normally do not harbor chromaffin tissue such as the liver, lungs, and bones.^3–5^ Although the histology of the resected primary paraganglioma may be somewhat helpful in predicting risk of metastasis, there have been no reliable histological features to accurately predict the risk of metastasis of a paraganglioma. Risk factors of malignant behaviors of paraganglioma include large tumor size and SDHB (succinate dehydrogenase B) mutation, but it is not clear if the two factors are independent of each other.^4,5,12^ Our patient indeed has a large primary paraganglioma and his young age of onset, tumor location, and the noradrenergic biochemical profile are all consistent with the presence of SDHB mutation, even though he declines genetic testing. The bone is the most common site of paraganglioma metastasis.^3–5,13^ Over half of patients with bony metastasis can have severe consequences such as severe pain and pathological fracture, as happened in our patient.^13^ Radiation therapy is the most common treatment.

Most metachronous metastases occur within several years after the initial diagnosis of paraganglioma.^5^ Late paraganglioma metastasis cases have been reported, mostly in patients with bony metastasis.^6–9,13^ To our knowledge, our patient has one of the longest gaps, 52 years, between the initial diagnosis and metastasis. The mechanisms responsible for the long dormancy of some metastatic paragangliomas remain unclear. Studies based on common cancers demonstrate that hematogenous bony metastasis occurs early when the primary cancer is still present but the metastatic malignant cells in the bone could be dormant for months to decades.^14^ Changes in chromatin structure and microenvironment may awaken the dormant malignant cells. In our patient, metastasis to the left femur possibly had occurred before the resection of the primary paraganglioma but remained dormant for over 50 years before being awakened. Should he had undergone regular surveillance, his avulsion fracture could have been prevented by early interventions such as radiation therapy.

## Conclusion

Very late paraganglioma metastasis can occur and result in severe consequences. Paragangliomas have significant malignant potential and require life-long surveillance for metastasis or recurrence.
